# Multifunctional Anti-Alzheimer’s Disease Effects of Natural Xanthone Derivatives: A Primary Structure-Activity Evaluation

**DOI:** 10.3389/fchem.2022.842208

**Published:** 2022-05-11

**Authors:** Xiaoyu Hu, Chan Liu, Kaichun Wang, Lanxue Zhao, Yu Qiu, Hongzhuan Chen, Jiangmiao Hu, Jianrong Xu

**Affiliations:** ^1^ Academy of Integrative Medicine, Shanghai University of Traditional Chinese Medicine, Shanghai, China; ^2^ Department of Pharmacology and Chemical Biology, Shanghai Jiao Tong University School of Medicine, Shanghai, China; ^3^ Joint International Research Laboratory of Ethnomedicine of Ministry of Education and Key Laboratory of Basic Pharmacology of Ministry of Education, Zunyi Medical University, Zunyi, China; ^4^ Institute of Interdisciplinary Integrative Medicine Research, Shanghai University of Traditional Chinese Medicine, Shanghai, China; ^5^ State Key Laboratory of Phytochemistry and Plant Resources in West China, Kunming Institute of Botany, Chinese Academy of Sciences, Kunming, China

**Keywords:** α-mangostin, Alzheimer’s disease, multifunctional, structure-activity, neuroinflammation, amyloid beta

## Abstract

**Background:** A series of α-Mangostin (α-M) derivatives were designed and synthesized. α-M and four analogues were evaluated for their multifunctional anti-Alzheimer’s disease (anti-AD) effects on fibrillogenesis, microglial uptake, microglial degradation, and anti-neurotoxicity of Aβ, as well as LPS-induced neuroinflammation. The differences in bioactivities were analyzed to understand the structure-activity relationship for further modifications.

**Purpose:** This study aims to investigate the anti-AD effects of α-M and elucidate its structure-activity relationship by comparing difference between α-M and several analogues.

**Methods:** Aβ fibrillogenesis was detected by Thioflavin T fluorometric assay. The levels of Aβ_1-42_ and inflammatory cytokines were evaluated by enzyme-linked immunosorbent assay. Neuron viability was examined by the CCK-8 assay. The morphology of ZO-1 of bEnd.3 cultured in BV-2-conditioned medium was evaluated by immunofluorescence staining.

**Results:** Aβ fibrillogenesis was significantly inhibited by co-incubation with α-M, Zcbd-**2** or Zcbd-**3**. α-M, Zcbd-**2**, Zcbd-**3,** and Zcbd-**4** decreased the levels of Aβ_1-42_ and inflammatory cytokines, and promoted Aβ uptake, degradation and anti-inflammation effects inflammation in microglia. α-M and Zcbd-**3** protected neuron viability from Aβ-induced neurotoxicity, and preserved tight junction integrity of bEnd.3 against LPS-induced neuroinflammation.

**Conclusion:** Zcbd-**3** acted as α-M almost in all effects. The structure-activity analysis indicated that the 3-methyl-2-butenyl group at C-8 is essential for the bioactivity of α-M, while modifying the double hydroxylation at the C-2 position may improve the multifunctional anti-AD effects.

## Introduction

Alzheimer’s disease (AD), a devastating and eventually fatal neurodegenerative disease, is characterized by progressive loss of cognition and disruption of essential functions (Scheltens et al., 2021). As the fifth leading cause of death worldwide (Karaman et al., 2020), AD affects more than 50 million people with a heavy emotional and financial burden on families and healthcare systems (Cortes-Canteli and Iadecola, 2020; Jia et al., 2020).

The progressive accumulation of Aβ plaques during the course of aging is one of the critical initiators of AD. In addition to the overproduction of Aβ extracellular deposition, the decline in Aβ clearance also results in cognitive impairment. In aging brains, the co-localization of microglia and Aβ plaques is usually found. As the central nervous system resident immune cells, microglia play a crucial role in uptake of excessive misfolded proteins and cell debris (Leng and Edison., 2021). The loss of Triggering receptor expressed on myeloid cells 2 (TREM2), a phagocytosis-mediated recoptor in microglia is closely linked to the risk of AD (Ulland et al., 2017). Different from classical phagocytosis mediated by the IgG complement receptors, Aβ was reported to interact with microglia by forming a cell surface complex including CD36, CD47, and α_6_β_1_ integrin (Koenigsknecht and Landreth., 2004). Moreover, pattern recognition receptors such as toll-like receptors, and integrin and scavenger receptors across microglia cell membrane recognize various Aβ species and induce microglia phenotypic alternation by activating phagocytic or inflammatory pathways. The downstream signal transduction pathways lead to cytoskeleton rearrangement, engulfment of Aβ and production of pro-inflammatory factors (Sengupta et al., 2016).

After phagocytosis, the degradative function of microglia is relatively time consuming. Aβ binds to the phagocytosis receptors in the surface of the microglial membrane. Then phagocytic cup is formed through membrane invaginations. These invaginated membranes extend and close up into single-membrane phagosomes containing Aβ, which subsequently fuses with lysosomes to form phagolysosome and is degraded finally. BECN-1, ATG-7, LC3 and other autophagy modulators have been reported to participate in this process, which suggests high functional similarity between these two degradation pathway (Plaza-Zabala et al., 2017).

However, not all Aβ is deposed unimpededly. Researches indicated that microglia tend to phagocytose fibrils rather than oligomers. In addition, Aβ oligomers are more neurotoxic and thought to be directly responsible for neurodegeneration. These spheres are 3–10 nm in diameter. They bind cell surfaces to disrupt the functions of a couple of receptors, including NGF receptors, NMDA receptors, insulin receptors, frizzled receptors, and so on (Kayed and Lasagna-Reeves., 2013). They also increase membrane permeabilization, induce loss of ion homeostasis, mitochondrial dysfunction, oxidative stress, disturbance of autophagy, and inflammatory response (Doens and Fernández., 2014). These pathologic cellular processes inevitably lead to cell death and neurodegeneration such as AD.

Due to the complex and multiple etiology and pathogenesis of AD, which is comprised of amyloid-β (Aβ) aggregation (Busche and Hyman, 2020), microglia-mediated neuroinflammation (Wang et al., 2017), increased oxidative stress, and impaired neurotransmission (Drummond et al., 2020), single-target drugs are inadequate to achieve therapeutic effects. The limited clinical efficacy on AD progression has shifted the focus from single-target drugs to multifunctional drugs, which are more beneficial and effective for treating complex diseases, such as AD (Benek et al., 2020). Multifunctional drugs possess pleiotropic pharmacologic actions and display various advantages such as enhanced therapeutic efficacies, improved safety profiles and reduced side effects (Wenzel and Klegeris, 2018; Wang et al., 2019; Uddin et al., 2021). More and more research on multifunctional drugs for AD was undertaken in the past decade (Zhang et al., 2019).

As important resources for the exploration of lead compounds with potential bioactivities, natural products have attracted increasing attention. Over 40% of approved molecules by the United States Food and Drug Administration are derived from natural products or their derivatives (Newman and Cragg., 2016). Some natural products have shown multifunctional anti-AD effects. For example, Resveratrol, a polyphenolic phytoalexin from medicinal plants, grapes, peanuts, and red wines, decreases reactive oxygen species (3), inhibits Aβ_1-42_ aggregation and exerts neuroprotective effects (Li et al., 2014). A series of natural products like hesperidin (Chakraborty et al., 2016), matrine (Cui et al., 2017), tenuazonic acid (Poliseno et al., 2021) and licochalcone (Liu et al., 2021) display therapeutic potentials of AD by participating in two or more regulative means, including acetylcholinesterase (AChE) inhibition, metal chelation and anti-neuroinflammation.

Mainly, α-Mangostin (α-M) is a polyphenolic xanthone derived from mangosteen. Recent studies demonstrated that α-M has a broader range of pharmacological properties, such as anti-cancer (Chitchumroonchokchai et al., 2013), antioxidant (Shen et al., 2014), anti-obesity, neuroprotective, hepatoprotective, and cardioprotective activities. Based on its versatile bioactivities, whether it affects neurodegenerative diseases arouses broad interests. It was found that α-M inhibited microglia-mediated neuroinflammation and neurotoxicity by targeting NF-κB and NADPH oxidase (Hu et al., 2016) and reversing the ROS overproduction, the activation of caspase cascade, and the dysfunction of mitochondria (Hao et al., 2017). In addition, its ability to scavenge Aβ in the hippocampus was validated in both the computational network pharmacological analysis and AD model rats (Chen et al., 2020). In the previous study (Zhao et al., 2017), we found that α-M could disassemble Aβ oligomers, inhibit Aβ fibril formation, enhance Aβ uptake and degradation in microglia cells and alleviate LPS-induced microglial activation and neuroinflammatory response to delay the AD progression. In addition, it decreased amyloid deposition in AD model mice, leading to the amelioration of neurologic changes and the cognition improvement in behavioural tests (Wang et al., 2012; Yao et al., 2016; Guan et al., 2020). Although α-M has been proved to be a potential candidate to treat AD, the intracerebral efficacy was limited by its poor penetration across the blood-brain barrier. Although we showed that encapsulation of PEG-PLA nanoparticles could improve the biodistribution of α-M *in vivo*, other alternative strategies should be explored to overcome this issue (Yao et al., 2016).

**FIGURE 1:**
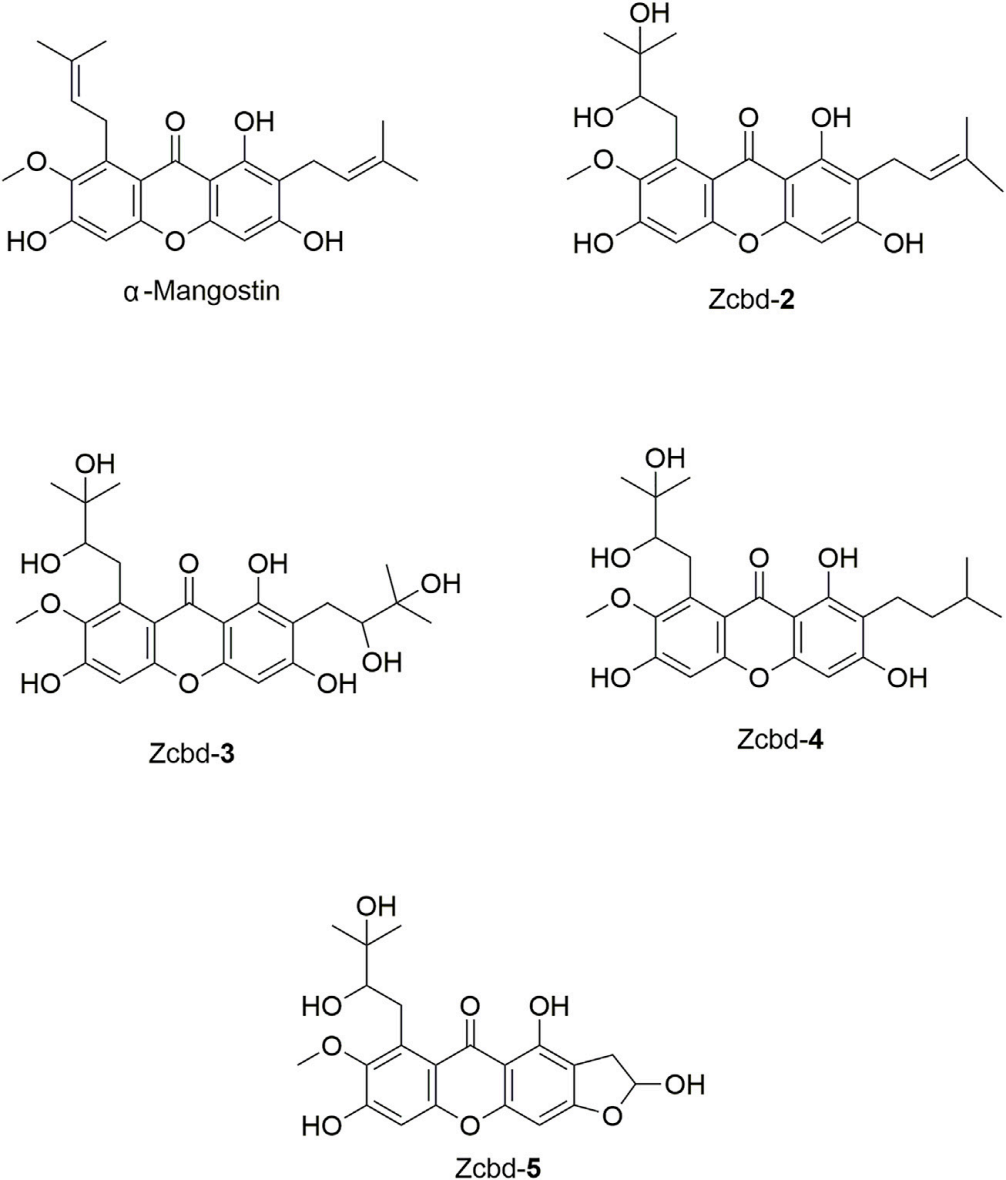
The chemical structure formulas of α-Mangostin (α-M), Zcbd-**2**, Zcbd-**3**, Zcbd-**4** and Zcbd-**5**. Molecular formula: C_24_H_26_O_6_ (α-M), C_24_H_28_O_8_ (Zcbd-**2**), C_24_H_30_O_10_ (Zcbd-**3**), C_24_H_30_O_8_ (Zcbd-**4**), and C_21_H_22_O_9_ (Zcbd-**5**). Molecular weight: 410.47 (α-M), 444.48 (Zcbd-**2**), 478.49 (Zcbd-**3**), 446.50 (Zcbd-**4**), and 418.40 (Zcbd-**5**).

Structural modification has been proved to be a valuable approach to deal with the deficiencies such as complex structures, metabolic instability, and low solubility (Yao et al., 2017). Our study demonstrated the limited *in vivo* application of α-M due to its hydrophobic scaffold, which leads to poor aqueous solubility and low bioavailability in the brain. Therefore, structure modification is inevitable to enhance the druggability of α-M. The structure optimization is based on knowledge about the respective influences of key modification sites on their multifunctional activities. However, only a little was known about it. To elucidate the structure-function relationship of α-M’s anti-AD activities, we investigated the effects of α-M and several representative analogues (Figure 1) on the aggregation and the clearance of Aβ, the anti-neurotoxicity, and neuroprotection from LPS-induced inflammatory response and tight junction impairment. Our data demonstrated that the performances of the five derivatives varied on inhibiting the formation of Aβ fibrils, promoting Aβ uptake and degradation in microglia, alleviating LPS-induced neuroinflammatory response as well as rescuing Aβ oligomer-induced neurotoxicity, which identified potential sites for α-M modification without activity loss on multifunctions.

## Materials and Methods

### Materials

Aβ_1-40_ and Aβ_1-42_ were purchased from Sigma (St. Louis, MO, United States) and dissolved in hexafluoroisopropanol (Sigma-Aldrich Corporation, St. Louis, MO, United States) at 1 mg/ml, stored at −20°C. α-M was purchased from Shanghai Sunny Biotech Co. Ltd., with a purity >99% by HPLC and dissolved in DMSO to 5 mM, stored at −20°C. The xanthone derivatives were synthesized as our previously published method (Chi et al., 2018). Cell counting kit-8 (CCK-8) was purchased from Dojindo Laboratories (Kumamoto, Japan). LPS (*Escherichia coli* 0111:B4) and dimethylsulfoxide (DMSO) were purchased from Sigma-Aldrich (St. Louis, MO, United States). Dulbecco’s modified Eagle’s medium (DMEM), minimal essential medium (MEM), fetal bovine serum (FBS), Neurobasalmedium, Gluta-MAX™-I supplements, non-essential amino acids and B27 supplement were purchased from Gibco (Grand Island, NY, United States). Penicillin, streptomycin, phosphate-buffered saline (PBS, pH 7.4), and other cell culture reagents were purchased from HyClone Laboratories (Logan, UT, United States). DAPI and BCA kits were purchased from Beyotime Biotechnology (Shanghai, China). Rabbit anti-Zona Occluden-1 (ZO-1) antibodies were purchased from Abcam (Cambridge, MA, United States). Alexa Fluor 647-conjugated goat anti-rabbit IgG was from Abcam (Cambridge, CA, United States). Aβ ELISA kits were purchased from Invitrogen (Thermo Scientific, PA, United States). TNF-α and IL-6 ELISA kits were purchased from Multisciences Biotech (Hangzhou, China).

### Cell Culture

With mild modifications, primary mouse cerebral cortical neurons were prepared as previously described (Kaech and Banker, 2006). Neurons were dissected from C57BL/6 mice at postnatal day 1 (Sprague-Dawley, Slac Laboratories, Chinese Academy of Sciences). The embryos were collected, and the cortices were dissected in ice-cold Hank’s buffered salt solution without Ca^2+^ and Mg^2+^ (GIBCO, Grand Island, NY, United States). Dissected pieces of cortical tissue were pooled together and transferred to an enzymatic dissociation medium containing 0.05% trypsin-EDTA (Gibco, Grand Island, NY, United States) and then incubated for 6–8 min at 37°C. After enzymatic dissociation, the trypsin solution was aspirated, and the tissue was triturated with a sterile Pasteur pipette in complete Minimum Essential Medium (MEM, 10% FBS (v/v), 5% horse serum (v/v), 2 mM L-glutamine, 100 units/mL penicillin, 100 mg/ml streptomycin) plus 2000 IU/ml DNase. After 800 g for 8 min centrifugation, the pellets were resuspended in complete MEM medium and plated on poly-L-lysine pre-coated 96-well or 24-well plates. Cells were cultured in a humidified atmosphere (5% CO_2_—95% room air) at 37°C for 2 h, then the medium was replaced with neurobasal medium plus 2% B27 and 2 mM Gluta-MAX™-I supplements. The purity of neurons is around 90%, as indicated by Hoechst staining for nucleus and β-tubulin staining for neural cell body and neurites.

The murine microglial cells BV-2 (3111C0001CCC000063) was obtained from the National Platform of Experimental Cell Resources for Sci-Tech (4), and cultured in DMEM supplemented with 10% FBS, 100 U/mL penicillin, 100 μg/ml streptomycin, 1% Gluta-MAX™-I supplements and 1% non-essential amino acids. The murine mouse brain endothelial cell line bEnd.3 (ATCC CRL2299) was obtained from ATCC and cultured in DMEM with 0.45% glucose, 0.37% NaHCO_3_, 4 mM glutamine, 10% FBS, 100 μg/ml penicillin, and 100 μg/ml streptomycin. Cells were grown in a humidified atmosphere containing 5% CO_2_ at 37°C and passaged every 2–3 days. When reaching the required confluence, cells were changed to serum-free medium prior to drug treatment.

### ThioflavinT Fluorescence Assay

The fibril formation of the Aβ peptides was measured by Thioflavin T (ThT) (Sigma, St. Louis, MO, United States) fluorometric assay according to published reports with some modifications (LeVine, 1993; Dai et al., 2010). Aβ was dissolved at 1 mg/ml in ddH_2_O, the α-M stock was diluted in 0.1 M TBS with 0.02% Tween 20, and ThT was dissolved in 50 mM Glycine-NaOH (pH 8.5).

To evaluate 24 h-inhibition of Aβ fibril formation by α-M, Zcbd-**2**, Zcbd-**3**, Zcbd-**4**, and Zcbd-**5**, Aβ_1-40_ (1 μg/ml) was mixed with α-M or its analogues in 1:1 molar ratio and incubated for 30 min. After incubation, ThT was added to each test sample to a final concentration of 10 μM and set at 37°C. The ThT fluorescence was monitored at an excitation of 446 nm and an emission of 485 nm with a bandwidth of 5 nm, and the measurement was continued for 24 h at 30 min intervals using Varioskan Flash plate reader (Thermo Scientific, PA, United States).

To monitor the disaggregation of preformed fibrils, Aβ_1-40_ (100 mg/ml) was incubated for 2 days at 37°C to generate fibrils. Then the preformed fibrils were mixed with an equal molar amount of α-M or its analogues for 8 h at 37°C. After incubation, ThT was added to each test sample to a final concentration of 10 μM. The ThT fluorescence was monitored at an excitation of 446 nm and an emission of 485 nm with a bandwidth of 2.5 nm, and the measurement was continued for 10 min at 1 min intervals using Varioskan Flash plate reader (Thermo Scientific, PA, United States).

### Aβ Uptake and Degradation Assay

BV-2 cells were pre-treated with α-M (250 nM) or its analogues (100 nM) for 24 h. Then the BV-2 cells were incubated for an additional 4 h in DMEM with a mixture of 1 μg/ml Aβ and α-M (250 nM) or its analogues (100 nM). Afterwards, cells were extensively washed with PBS and were lysed in cell lysis buffer (50 mM Tris-HCl, pH 7.4, 150 mM NaCl, 5 mM EDTA, 1% SDS) supplemented with EDTA-free protease inhibitors (Roche). Lysates were centrifuged at 12,000 g at 4°C for 15 min. Supernatants were collected and further applied for ELISA. Aβ levels were measured using Aβ ELISA kit (Wako) and normalized to total protein concentration assessed by the BCA method (Pierce).

### Measurement of Tumour Necrosis Factor-α and Interleukin-6

BV-2 cells were pre-treated with α-M (250 nM) or its analogues (100 nM) for 2 h and then stimulated by LPS (100 ng/ml) for 24 h. Then the medium from each group was collected. According to the manufacturer’s instructions, the concentrations of TNF-α and IL-6 in the cell culture media were determined using commercially available ELISA kits (MultiSciences, Hangzhou, China). The concentration was calculated from a standard curve obtained by adding sodium nitrite to DMEM.

### Anti-neurotoxicity Induced by Aβ_1-42_ Oligomers

For the preparation of Aβ_1–42_ oligomer, the dried monomeric peptide Aβ was resolved in ddH_2_O at a concentration of 100 mM and incubated at 4°C for 24 h. The cell viability of primary cultured cerebral cortical neurons with Aβ oligomers or α-M/Zcbd-**3** was measured by the CCK-8 kit (Dojindo, Kumamoto, Japan). Cells were seeded onto 96-well plates at a density of 1 × 10^4^ cells per well and allowed to grow to the required confluence. On day 7 after culturing, Aβ_1-42_ oligomers and α-M/Zcbd-**3** were added directly to reach the final concentrations of 1 μM for Aβ_1-42_ oligomers and 0.5, 5, and 50 nM for α-M/Zcbd-**3**. Then the cells were incubated for 24 h, and cell viability was measured with a CCK-8 kit according to the manufacturer’s instructions. Cells were incubated at 37°C for 1 h; the absorbance was read by Varioskan Flash plate reader (Thermo Scientific, PA, United States) at 450 nm.

### Detection of the Expression of ZO-1

Mouse brain endothelial bEnd.3 cells were seeded onto 35-mm dishes at a density of 2 × 10^5^ per dish. After treatment, they were fixed with 4% paraformaldehyde for 1 h, permeabilized by 0.4% Triton X-100 in PBS, and blocked in PBST supplemented with 5% sheep serum for 30 min at room temperature. Afterwards, they were incubated with rabbit anti-ZO-1 (1: 400) antibody overnight at 4°C. After PBS washing, they were incubated with an Alexa Fluor 647-conjugated goat anti-rabbit secondary antibody (1:1000) for 1 h at room temperature. Then cells were counterstained with DAPI for 10 min. Images were captured with a Leica TCS SP8 confocal microscope (Leica, Wetzlar, Germany) and further analyzed using Image J and Adobe Photoshop CC 2019.

### Data Analysis and Statistics

Data were presented as mean ± standard error of the mean (SEM). They were analyzed by student’s T-test or one way-ANOVA followed by Dunnett’s test using GraphPad Prism 8 statistical analysis software, with *p* < 0.05 taken to indicate significant difference.

## Results

### α-Mangostin and its Analogues Inhibit Aβ Fibril Formation

**FIGURE 2:**
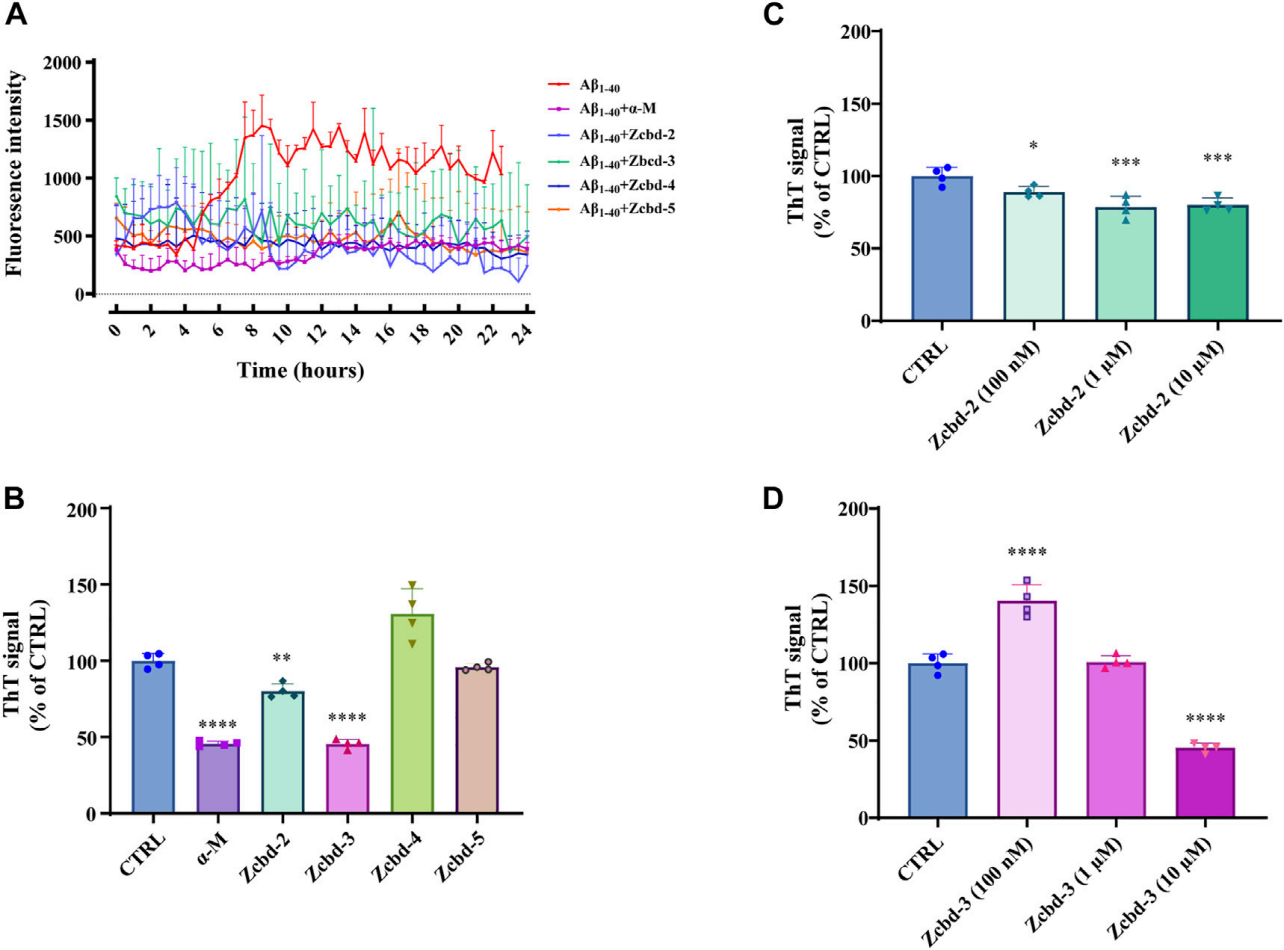
α-M and its analogues inhibited Aβ fibrillogenesis **(A)** Inhibitory effects of α-M and its analogues effect on Aβ fibril formation 24 h kinetic detection. 10 μM of ThT was incubated with Aβ_1-40_ or the mixture of Aβ_1-40_ and α-M or its analogues at 37°C. The newly formed fibrils were measured by a 24 h continuous ThT fluorescence at 30 min intervals **(B)** Disaggregation of preformed Aβ fibrils by α-M and its analogues. Aβ_1-40_ was firstly incubated at 37°C for 48 h. Then α-M or its analogues were added and incubated for another 8 h. ThT was subsequently added into the mixture with a final concentration of 10 μM. The fluorescence was monitored with excitation at 446 nm and emission at 490 nm **(C)** and **(D)** Dose-effect relationship in the disaggregation of preformed Aβ fibrils by Zcbd-**2** and Zcbd-**3**. Zcbd-**2** or Zcbd-**3** (100 nM, 1 μM, and 10 μM) was added to preformed Aβ fibrils and incubated for additional 2 days. Then ThT fluorescence was detected. All data represent mean ± SEM from 4 independent experiments. ∗*p* < 0.05, ∗∗*p* < 0.01, ∗∗∗*p* < 0.001, ∗∗∗∗*p* < 0.0001 compared to the control group (CTRL).

To analyze the kinetic inhibition on Aβ fibrillogenesis, a 24 h kinetic ThT fluorescence assay was performed to detect the dynamic level of Aβ fibril formation. By binding to fibrillar structures rather than monomeric amyloid, a shift was produced in the emission spectrum of ThT, thereby generating a fluorescent signal, which is directly proportional to the amount of fibrils formed. The examination of ThT demonstrated that the fluorescence density sharply increased after 8 h of incubation with Aβ and then steadily fluctuated between 1,000 and 1,500 within the 24-h detection. In contrast, the fluorescence density of Aβ_1-40_ co-incubated with α-M or its analogues all kept at a low level, which indicated that α-M and its analogues significantly inhibited the formation of Aβ_1-40_ fibrils (Figure 2A).

To measure the effects of α-M and its analogues on fibril disaggregation, Aβ_1-40_ was pre-aggregated for 48 h at 37°C before incubated with equal molar α-M or its analogues for another 8 h. Then endpoint ThT fluorometric assay was conducted to measure the disaggregation effects of the five compounds on Aβ fibril formation. The fluorescence signal density (Figure 2B) showed that the disaggregation of pre-aggregated fibrils was induced to 54.32%, 19.86%, and 54.58% of that co-incubated with α-M, Zcbd-**2** and Zcbd-**3**, respectively. At the same time, Zcbd-**4** and Zcbd-**5** could not decrease the ThT signal.

Based on the significant effects of Zcbd-**2** and Zcbd-**3**, we further observed the disaggregation of Aβ fibrils in the co-incubation with Zcbd-**2** and Zcbd-**3** at three concentrations (100 nM, 1 μM, and 10 μM). As shown in Figure 2C, the ThT signal was attenuated by 11.00%, 21.44%, and 19.86% after treatment of Zcbd-**2** at a concentration of 100 nM, 1 μM, and 10 μM. In Figure 2D, a decrease of the ThT signal was also displayed after treatment of Zcbd-**3**, confirming that both Zcbd-**2** and Zcbd-**3** could induce the disaggregation of pre-aggregated Aβ_1-40_. In addition, the results in Figure 2C demonstrated that the ThT signal was further weakened with the increasing concentration of Zcbd-**2**, indicating that Zcbd-**2** possessed a dose-dependent promotion on Aβ fibrils disaggregation. In contrast, low concentrations of Zcbd-**3** kept little inhibitory effect on Aβ aggregation, yet it displayed a 2.75-fold higher disaggregation efficacy than Zcbd-**2** at a high concentration of 10 μM (Figure 2D).

### α-Mangostin and Zcbd-3 Promote Aβ_1-42_ Uptake and Degradation by Microglial Cells

**FIGURE 3:**
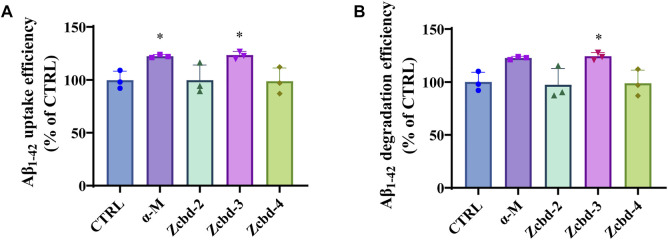
α-M and its analogues enhanced Aβ uptake and degradation in microglia BV-2 cells **(A)** The enhancement of Aβ uptake in microglia by α-M or its analogues. Microglia BV-2 cells were pre-treated with α-M (250 nM) or its analogues (100 nM) for 24 h. Then Aβ (1 μg/ml) was added into the medium for co-incubation. After 4 h incubation at 37°C, the medium was collected to extract the supernatant by centrifugation. The amount of Aβ in the supernate was detected **(B)** The acceleration of Aβ degradation in microglia by α-M or its analogues. BV-2 were pre-treated with α-M (250 nM) or its analogues (100 nM) for 24 h. Then Aβ was added into the medium for co-incubation. After 4 h incubation at 37°C, both the medium and the cell suspension were collected. Moreover, the amount of Aβ in the medium and cell lysate was detected, respectively. The degradation of Aβ was evaluated by measuring the difference between the initial total Aβ and the rest Aβ both in the medium and lysis of BV-2. All data represent mean ± SEM from 3 independent experiments. ∗*p* < 0.05 compared to the control group (CTRL).

AD is characterized by excessive Aβ deposition, which is widely recognized to result from an imbalance between its production and clearance. Aβ can be eliminated from the brain by enzymatic degradation and cellular uptake. As the primary cells involved in intracerebral Aβ clearance, microglia could gather around Aβ plaques and clear them by phagocytosis. Here, we detect the effects of α-M and its analogues on Aβ_1-42_ uptake and degradation in microglial cells by exposing Aβ_1-42_ to BV-2 following treatment with α-M or its analogues. The determination of the decreasing content of Aβ_1-42_ in the culture medium of BV-2 after 8 h co-incubation showed that α-M and Zcbd-**3** increased Aβ_1-42_ uptake efficiency by 22.70% and 23.71% (Figure 3A), respectively, which highlighted these two compounds could accelerate Aβ_1-42_ uptake of microglia.

Then we evaluated the degradation of Aβ_1-42_ by measuring the difference between the initial total Aβ and the rest Aβ both in the medium and lysis of BV-2. Both α-M and Zcbd-**3** promoted the Aβ_1-42_ degradation efficiency of BV-2 by 22.70% and 24.30%, respectively (Figure 3B). The results indicated that the structural modification in Zcbd-**3** would not impair the microglial clearance of Aβ by α-M. Meanwhile, the detection of Aβ_1-42_ in BV-2 lysis manifested that there was little Aβ residue in BV-2, suggesting that α-M and Zcbd-**3** largely prompted the degradability of BV-2 on Aβ oligomers uptaken by themselves.

### α-Mangostin and its Analogues Attenuate LPS-induced Inflammatory Response in BV-2 Cells

**FIGURE 4:**
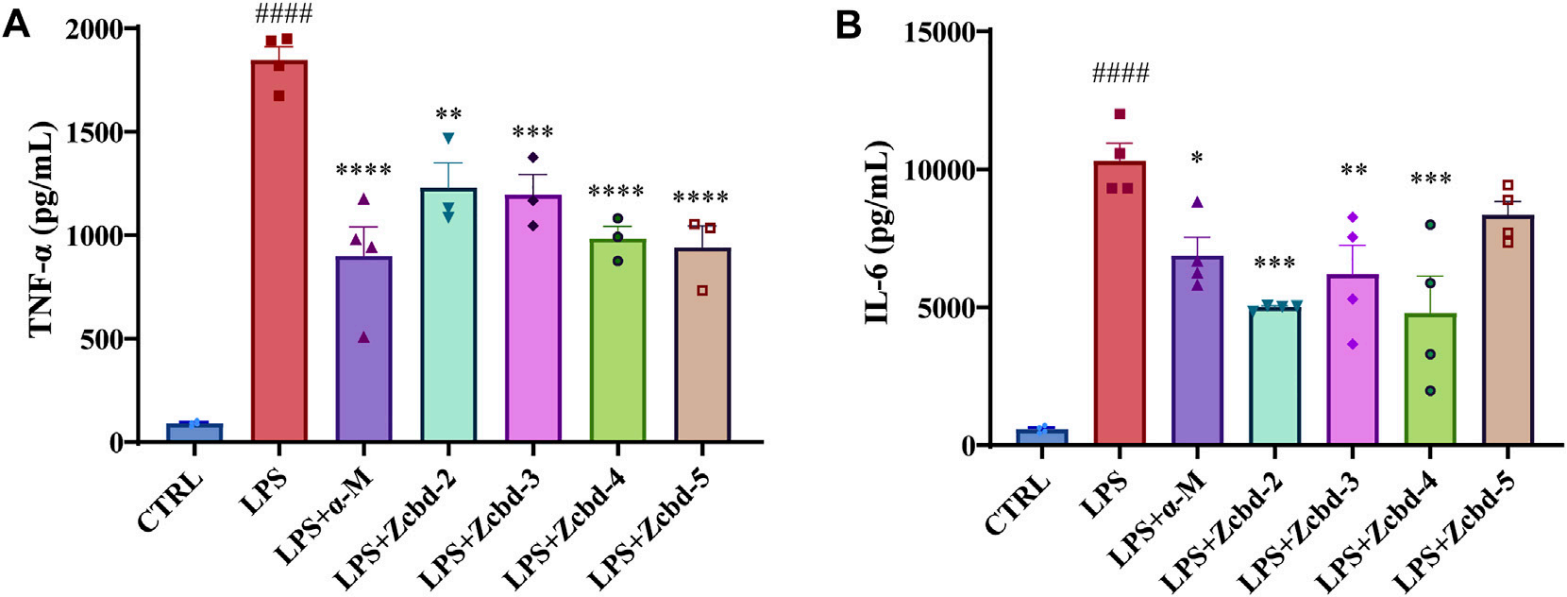
α-M and its analogues suppressed LPS-induced production of pro-inflammatory cytokines in microglia BV-2 cells. BV-2 cells were pre-treated with α-M or its analogues for 2 h prior to stimulation with LPS (100 ng/ml) for another 24 h. Then the protein levels of **(A)** tumour necrosis factor-α (TNF-α) and **(B)** interleukin-6 (IL-6) were measured by ELISA in LPS-stimulated BV-2. All data represent mean ± SEM from at least 3 independent experiments. ^∗^ *p* < 0.05, ^∗∗^ *p* < 0.01, ^∗∗∗^ *p* < 0.001, ^∗∗∗∗^ *p* < 0.0001 compared to the LPS-stimulated group (LPS); ####*p* < 0.0001 compared to the control group (CTRL).

TNF-α and IL-6 are two of the essential pro-inflammatory cytokines released by microglia under stress response. As shown in Figures 4A,B, the expression of TNF-α and IL-6 in BV-2 were drastically increased after LPS (100 ng/ml) stimulation (by 20.58 fold for TNF-α and by 18.15 fold for IL-6, respectively); while these effects were alleviated by α-M and its analogues. The Elisa assay demonstrated significant decreases in TNF-α and IL-6 of the culture medium after co-incubated with α-M, Zcbd-**2**, Zcbd-**3**, Zcbd-**4** or Zcbd-**5** by 51.30%, 33.37%, 35.21%, 46.76%, and 49.07% for TNF-α and 33.34%, 51.38%, 39.91%, 53.54%, and 19.04% for IL-6, respectively. All these analogues retained the anti-inflammatory activity of α-M. Zcbd-**4** and Zcbd-**5** exerted similar inhibitory effects on the expression of TNF-α as α-M; meanwhile, Zcbd-**2**, Zcbd-**3** and Zcbd-**4** performed better than α-M in inhibiting the expression of IL-6.

### α-Mangostin and Zcbd-3 Protect Mouse Cerebral Cortical Neurons From Aβ_1-42_ Oligomers-induced Neurotoxicity

**FIGURE 5:**
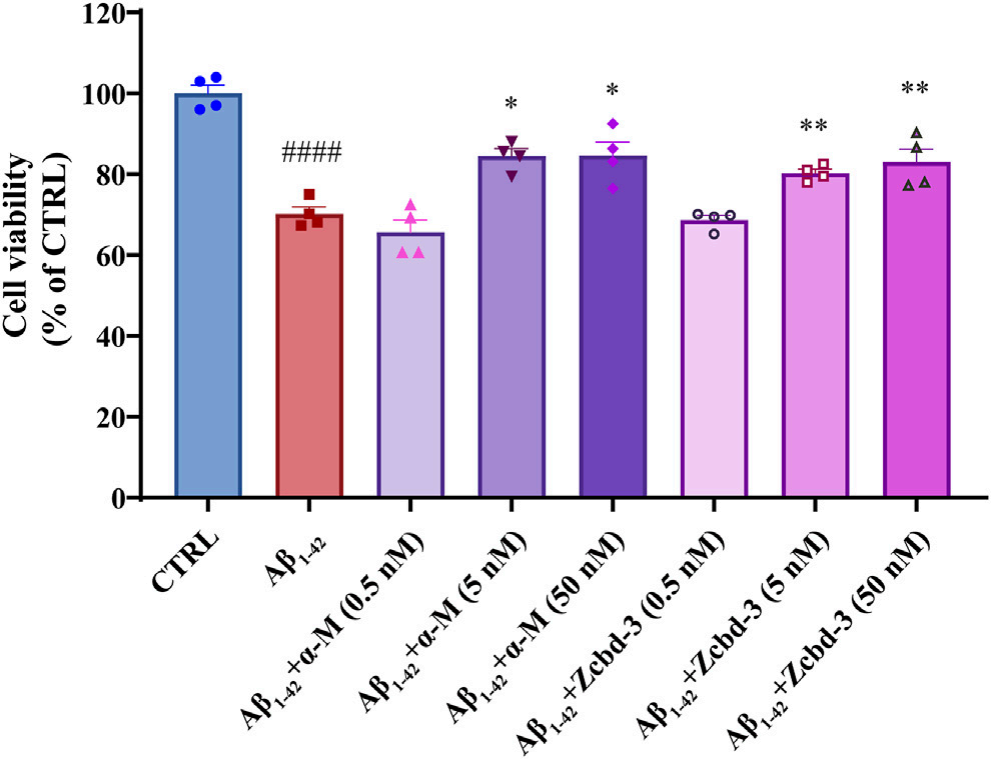
α-M and its analogues rescued the Aβ_1-42_ induced neurotoxicity in primary cultured cerebral cortical neurons from C57BL/6 mice. Primary cultured mouse cerebral cortical neurons were incubated with Aβ_1-42_ oligomers (1 μM) and various concentrations of α-M or Zcbd-**3** (0.5, 5, and 50 nM) for 24 h. Then the cell viability was measured by CCK-8 assay. All data represent mean ± SEM from 4 independent experiments. *p < 0.05, **p < 0.01 compared to the Aβ_1-42_-stimulated group (Aβ_1-42_); ####*p* < 0.0001 compared to the control group (CTRL).

Aβ oligomers have been confirmed to be neurotoxic through inducing dendritic simplification, synaptic dysfunction, dystrophic neurites and cell apoptosis. To precisely evaluate the protective effects of α-M and Zcbd-**3** on neurons exposed to Aβ_1-42_ oligomers, CCK-8 assays were performed on primary cerebral cortical neurons of fetal C57BL/6 mice to better simulate a brain situation. As shown in Figure 5, 24 h exposure to Aβ_1-42_ oligomers (1 μM) decreased cell viability by 29.83% of cortical neurons, while co-incubation of Aβ_1-42_ with 5 nM and 50 nM α-M significantly reversed the viability to 80.27% ± 1.69% and 83.02 ± 5.54%, respectively. According to the analysis above, the EC_50_ of α-M’s neuroprotection from Aβ_1-42_ oligomers was 0.70 nM. Meanwhile, Zcbd-**3** of equal concentration also rescued the amount of living neuronal to 84.50 ± 3.13% and 84.61 ± 5.79%.

### α-Mangostin and Zcbd-3 Preserve Tight Junction in bEnd.3 Cells

**FIGURE 6:**
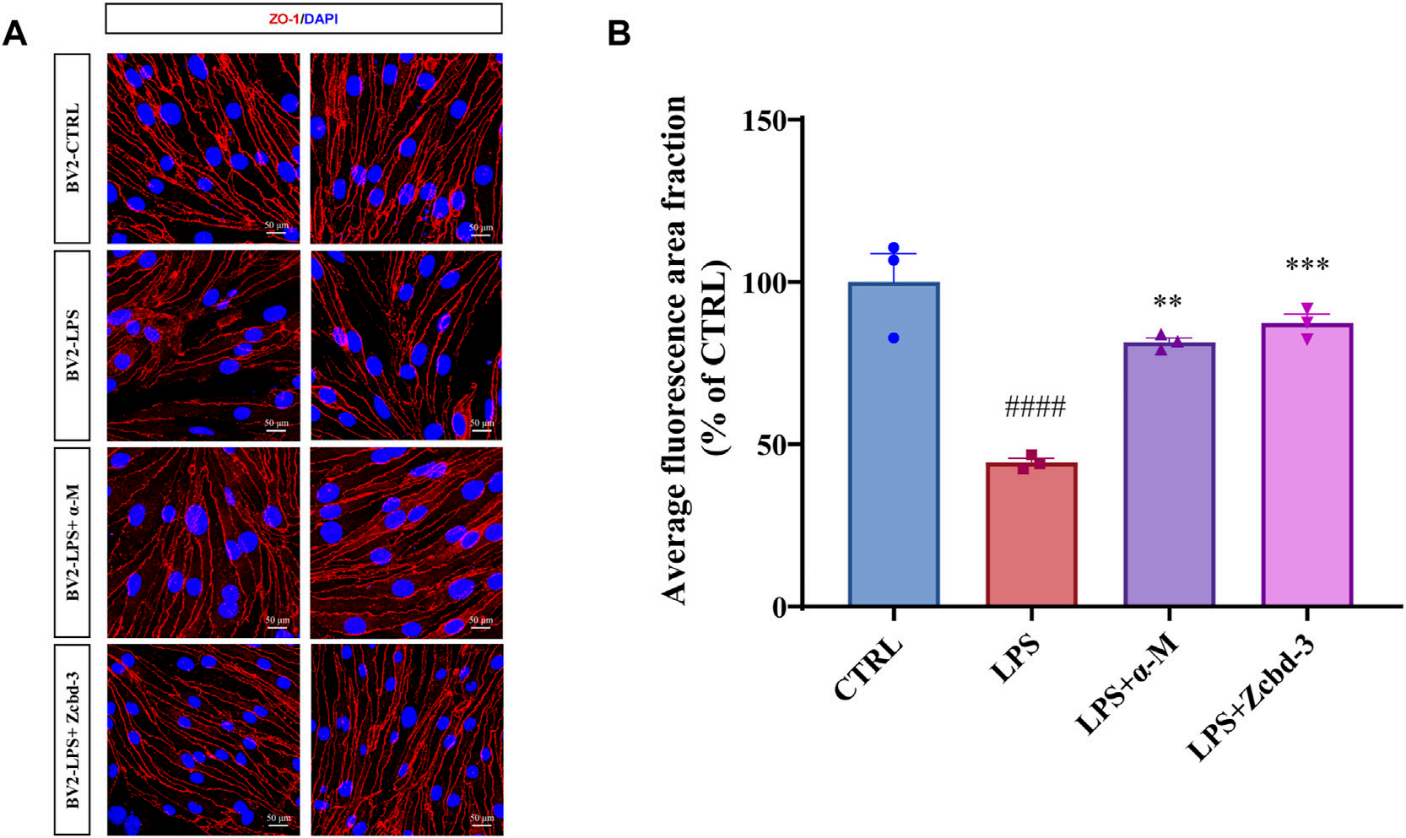
α-M and Zcbd-**3** ameliorated the alternation of tight junction (TJ) protein Zonula Occluden-1 (ZO-1) in LPS-stimulated bEnd.3 cells **(A)** α-M and Zcbd-**3** preserved the expression and location of ZO-1 localization in LPS-treated bEnd.3 cells. Mouse brain endothelial bEnd.3 cells were pre-treated with α-M or its analogues for 2 h prior to stimulation with LPS (100 ng/ml) for another 24 h. Then the cells were fixed and stained. The morphology and fluorescence intensity of ZO-1 was detected by a confocal laser-scanning microscope **(B)** The bar graph showed the quantitation of the average fluorescence area fraction in bEnd.3 cells. All data represent mean ± SEM from 3 independent experiments. **p < 0.01, ***p < 0.001, ****p < 0.0001 compared to the LPS-stimulated group (LPS); ####*p* < 0.0001 compared to the control group (CTRL). DAPI: blue, ZO-1: red, Scale bar: 50 μM.

Disruption of tight junctions (TJs) is a hallmark of AD, which enhances the permeability of BBB. Overactivated BV-2 by neuroinflammation may release cytokines to destroy the nervous system in a few ways, including BBB dysfunction. bEnd.3 mouse brain endothelial cell line keeps the characteristic of BBB as a functional barrier. Thus it is an ideal model for BBB research. In order to monitor an AD-anastomotic brain environment, BV-2 cells were stimulated by LPS (100 ng/ml, 24 h) to simulate inflammation response in AD patients’ brains. Then the BV-2-conditioned media was transferred to bEnd.3 cells to observe the effect of microglia-mediated neuroinflammation on BBB. As an intracellular scaffold protein, the numerical and spatial changes of ZO-1 reflect the integrity and functional status of BBB. Thus we detected the expression and distribution of ZO-1 in bEnd.3 mouse brain endothelial cells after 24 h-treatment with BV-2-conditioned media. The immunofluorescence staining results showed that the pretreatment with α-M or Zcbd-**3** before the administration of LPS remarkably rescued the expression of ZO-1 in bEnd.3 by 66.58% (α-M) and 77.31% (Zcbd-**3**), compared to the group without pretreatment. Meanwhile, as shown in Figure 6A, the spatial density, as well as the diameter and length of ZO-1, is remarkably improved by pretreatment with α-M or Zcbd-**3**. According to the increase in expression and distribution of ZO-1, we found that α-M and its analogue contribute to maintaining the morphological integrity of BBB affected by neuroinflammation. The data revealed that Zcbd-**3**, as well as α-M, possessed a strong inhibitory effect on the LPS-induced microglial neurotoxicity and exerted protection of brain structures.

## Discussion

The most widely accepted hypothesis for the pathogenesis of AD considers Aβ to play a crucial role in AD’s development and progression (Hardy and Selkoe, 2002; Polanco et al., 2018; Peng et al., 2020). A steady accrual of clinic data (Panza et al., 2019) supports the concept that a disorder between the production and clearance of Aβ is an early risk factor in AD (Lloret et al., 2019). Meanwhile Aβ levels are much higher in AD patients, and known to be closely associated with cognitive defects (Gouras et al., 2015; Bischof and Jacobs, 2019).

In AD, Aβ aggregates to form plaques and tangles, which comprise the histopathological hallmarks (Villemagne et al., 2018). Early evidence showed that Aβ fibrils, oligomers and protofibrils could diffuse into synaptic clefts by interacting with neuron membranes, and further initiate a cascade of events (Siddiqi et al., 2019), including progressive neuron loss, neural circuits disintegration, and neurological decline. Therefore, normal metabolism and maintenance of the homeostatic state of Aβ are essential for brain health (O'Brien and Wong, 2011).

Although the present clinical treatment could decrease the level of Aβ in blood and cerebrospinal fluid and clear Aβ deposits in the brain (Hung and Fu, 2017; Uddin et al., 2020), AD patients’ cognitive functions were not improved significantly. Due to AD’s complex pathophysiology comprising many intertwined subproblems, clinical trials of single-target drugs keep providing discouraging results. In this predicament, multifunctional drugs bring new opportunities in drug discovery and have appeared with more space. Natural products embody inherent structural complexity and biological activity (Das and Basu, 2017; Jin et al., 2019), which often leads to new targets, pathways, or modes of action and makes them important resources for multifunctional drugs of AD.

Natural products with diverse structures were pivotal sources for novel lead compounds in drug discovery. Our previous studies proved that α-M, one of the natural products, displayed protective effects on Aβ aggregation and inflammation-induced neurotoxicity. Because of its multifunctional anti-AD effects, α-M attracted our interest in anti-AD drug discovery. Nevertheless, several restrictions may hamper its extensive use, such as low solubility, absorption limitation, and metabolic stability. Structural modification is an inevitable way for α-M to achieve a final druggable candidate. Therefore, we designed a series of α-M analogues to understand better its structure-activity relationship, especially which part is applicable for modification.

In this study, we demonstrated the effects of α-M and its four analogues on inhibiting Aβ fibrillogenesis, disaggregating preformed Aβ fibrils, accelerating Aβ clearance, protecting neurons from LPS-induced neuroinflammatory response, anti-neurotoxicity, and preserving the integrity of tight junctions.

The ThT fluorescence assay results revealed that α-M and the analogues inhibited the formation of Aβ fibrils during 24 h co-incubation. In addition, two of α-M’s analogues, Zcbd-**2** and Zcbd-**3**, significantly disaggregated preformed Aβ. Pharmacodynamic correlation between dosage and activity is one of the prerequisites for satisfactory druggability. Thus we designed the co-incubation of Aβ and Zcbd-**2** and Zcbd-**3** of three different concentrations (100 nM, 1 μM and 10 μM) to investigate the disaggregation of Aβ by co-incubation with Zcbd-**2** and Zcbd-**3**. The fluorescence signals reflected that both Zcbd-**2** and Zcbd-**3** significantly affected the disaggregation of preformed Aβ fibrils in a concentration-dependant manner. The ThT results also suggested that all four derivatives have no negative influence on the Aβ fibrillogenesis inhibitory activity of α-M. Moreover, the modifications in Zcbd-**2** and Zcbd-**3** retained the disaggregating effect of α-M on preformed fibrils.

There are several widely-recognized Aβ clearance pathways in the central nervous system, including interstitial drainage, cerebrospinal fluid, and proteolytic digestion. As innate immune cells in central nervous system, microglia help maintain neural environment homeostasis, support regeneration and induce repair through clearing debris and aberrant proteins, including Aβ plaques (Harry, 2013). Any functional deficits in the microglia would have a severe impact on the brain’s health condition. Evidence proved that microglia in the aged brains (Angelova and Brown, 2019) were less evenly distributed and smaller with less ramification and dynamics. In addition, several works recently demonstrated that an exaggerated response with overexpression of pro-inflammatory signals or insufficient induction of anti-inflammatory factors is more likely to happen in aged brains due to the phenotypic shift and function decline in microglia (Rangaraju et al., 2018; Stephenson et al., 2018; Scheiblich et al., 2020). Therefore, triggering functionally regulation on aged microglia is one of the research approaches to alleviate pathological states through amending our immune function. In our study, α-M and Zcbd-**3** significantly elevated the efficiency of Aβ uptake and degradation in microglia, while Zcbd-**2** and Zcbd-**4** displayed little effect on the promotion of Aβ clearance. The results indicated that the variable sites in Zcbd-**3** were applicable for further structural modifications, meanwhile the structural changes in Zcbd-**2** and Zcbd-**4** may affect the ability of microglia to uptake and degrade Aβ.

Due to its larger surface area directly interacting with the neuronal membranes, it is now proposed that, compared to Aβ fibrils, Aβ oligomers are easier to diffuse into synaptic clefts, cause the impairment of neuronal signal transmittion, and result in the neurological decline characteristic of AD (Haass and Selkoe, 2007). Moreover, Aβ_1-42_ has been found to possess a greater propensity to aggregate compared to Aβ_1-40_, and it is the predominant component of AD plaques (Lin et al., 2019). It interacts initially with hydrophobic molecules in particular membrane lipids, further perturbs their structures, and causes actual holes that conduct ions and induce toxicity (Phillips, 2019). In the study mentioned above, we have investigated the influence on the function of Aβ and microglia. Besides, massive neuronal death, as a critical hallmark in the pathological process of AD (Selkoe, 2001), directly leads to severe clinical symptoms, including cognitive dysfunction, memory loss, and mood changes (Lazarov and Hollands, 2016). Thus alleviating Aβ_1-42_ oligomers-induced neurotoxicity is well-considered to be beneficial during such a slowly progressive and highly dynamic process in AD. To evaluate the protective effects of α-M and its analogues on Aβ-induced neurotoxicity, we treated α-M or its analogues-pretreated primary cerebral cortical neurons with Aβ_1-42_ oligomers. Cell viability after 24-h incubation was manifested by the CCK-8 assay, which demonstrated that co-administration of α-M or Zcbd-**3** significantly elevated the cell viability depressed by neurotoxic Aβ oligomers. The analysis results indicated that α-M and Zcbd-**3** remarkably attenuated the neurotoxicity of Aβ oligomers and exerted neuroprotective effects in cortical neuron cells.

In addition to Aβ deposition, microglia-mediated neuroinflammation also induces neuronal injury and memory dysfunction in AD (Colonna and Butovsky, 2017). Inflammation is a crucial contributor to CNS disorders such as PD and AD. It remains a debate whether inflammation is causative or secondary in AD because it is both a pathogeny for the disease development and a consequence of the disease progression. As a double-edged sword in AD, microglia acts in concert with astrocytes to cause neuronal injury (Subhramanyam et al., 2019). Excessive microglial activation is involved in initiating and amplifying immune response that ultimately worsens the neurodegenerative condition. A series of factors that mainly include pro-inflammatory cytokines, such as TNF-α and IL-6, are released by activated microglia (Hickman et al., 2018). They induced astrocytes into a neurotoxic state termed “A1” that leads to neuron cells’ death. Those A1 astrocytes are found mainly in CNS tissue from patients with neurodegenerative diseases, including AD (Hansen et al., 2018). To assess the effects of α-M and its analogues on microglia, we pre-treated BV-2 with α-M or its analogues for 24 h. Then LPS was added into the medium to induce an inflammatory response. It was observed that the pretreatment of α-M or its analogues could decrease the expression of TNF-α and IL-6 in LPS-induced neuroinflammation. Especially Zcbd-**4** displayed more potent effects on inhibiting the release of the pro-inflammatory cytokines than α-M, which suggested that the change in Zcbd-**4** itself is a modification to enhance the anti-inflammatory effect of α-M.

Microglia also works as the first line guardian against BBB injury in the central inflammation response (Thurgur and Pinteaux, 2019). BBB is a complex multi-cellular structure. It located at the interface to compart the central and peripheral nervous system, thus it is regarded as a sensor of homeostasis. So that any homeostasis disruption may result in BBB dysfunction. Researches have suggested that interaction between BBB and microglia dictates microglial to response to the dysfunction of neurovascular unit (NVU) (Liebner et al., 2018), which enables an integrated relationship between all components, including neuron cells, glia cells and BBB, to maintain a constant internal environment in our brains (Liu et al., 2020). On account of the critical roles of NVU and BBB in innate brain structure, the pathological conditions in AD are closely associated with defective barriers, which lead to the loss of their guard properties. The damage of the integrity of the BBB induced by Aβ is considered one of the critical factors leading to the occurrence and progression of AD (Salama et al., 2006; Cai et al., 2018; Sweeney et al., 2019). The unique BBB barrier function own to the tight junctions (TJs), which are closely packed between cerebral endothelial cells to hold a low paracellular permeability into the brain. ZO-1 is an intracellular scaffold protein that interacts with transmembrane proteins such as junctional adhesion molecules to maintain the integrity of TJs. Based on the protective effects of α-M and Zcbd-**3** on Aβ oligomers-induced neurotoxicity, we defected their effects on BBB dysfunction due to LPS-induced inflammatory response by evaluating the expression and morphology of ZO-1 in bEnd.3 cerebral microvascular endothelial cells. The immunofluorescence staining of ZO-1 illustrated that bEnd.3 pre-treated by α-M or Zcbd-**3** appeared to be closer to the normal status, which indicated the protective effects of both α-M and Zcbd-**3** on keeping the homeostasis of NVU.

**FIGURE 7:**
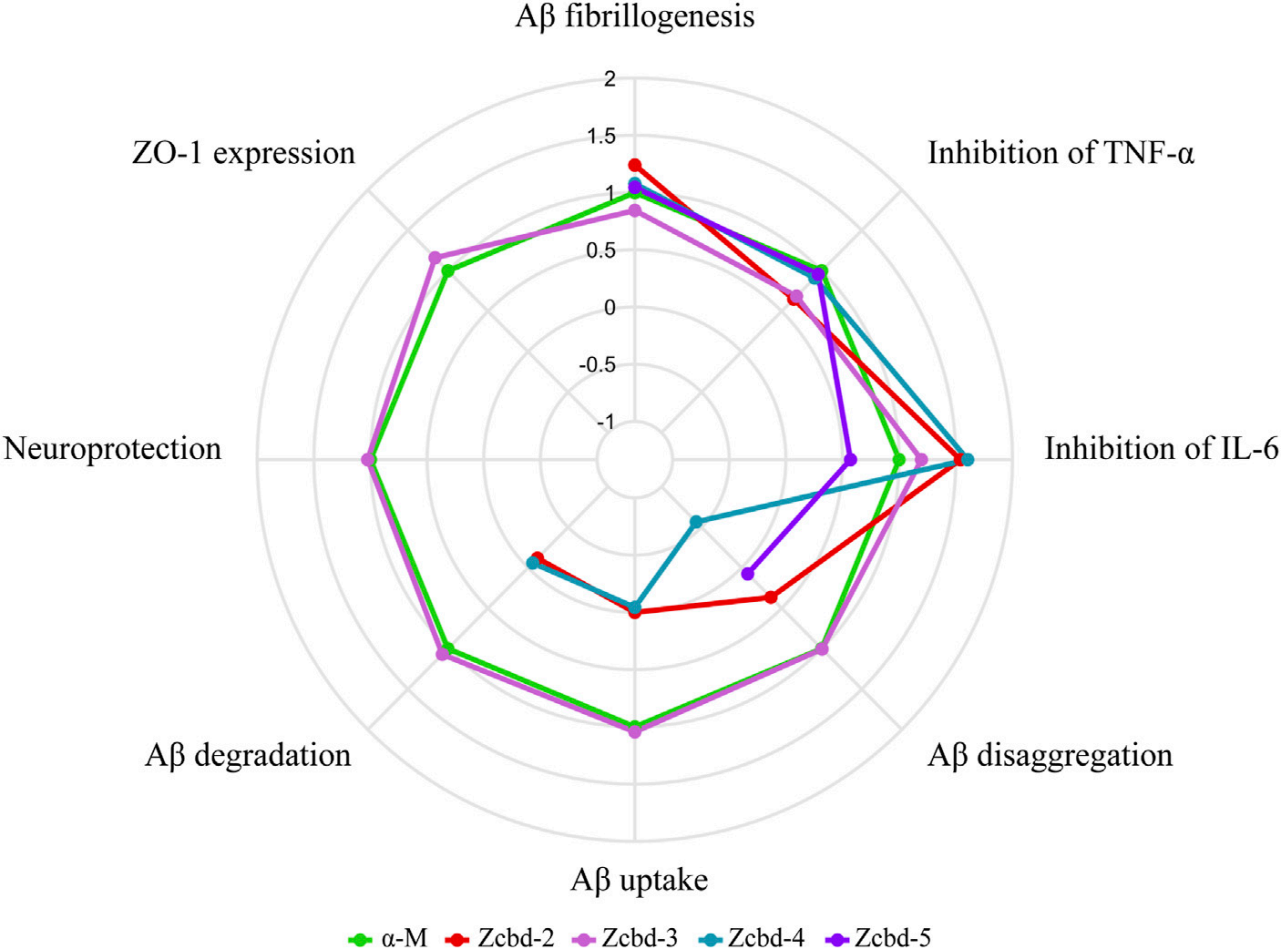
Multifunctional anti-AD effects of α-M and its analogues. The radar plot of properties included eight aspects, which were Aβ fibrillogenesis, inhibition of TNF-α, inhibition of IL-6, Aβ disaggregation, Aβ uptake, Aβ degradation, neuroprotection and ZO-1 expression. The performance of the four analogues was compared with the control group (CTRL) and normalized to the effects of α-M. Polylines were used instead of the value “0” to represent analogues’ performance, which was eliminated during the experimental process.

For α-M contains two 3-methyl-2-butenyl groups at C-2 and C-8 positions as potential functional groups, we synthesized four analogues to evaluate whether they are modifiable sites. These derivates share the same structural scaffold with α-M; meanwhile, the two 3-methyl-2-butenyl groups were modified differently. As shown in Figure 7, taking the performance of α-M as control, we evaluated each analogue by relative percentages in order to display all-round performance of each compound. The radar plot demonstrated that the effects of α-M and its analogues from eight aspects, which were Aβ fibrillogenesis, inhibition of TNF-α, inhibition of IL-6, Aβ disaggregation, Aβ uptake, Aβ degradation, neuroprotection and ZO-1 expression. According to Figure 7, α-M and Zcbd-**3** shared the hightest anti-AD activity in general; while Zcbd-**2** and Zcbd-**4** displayed weaker ability in lessening Aβ deposition, and Zcbd-**5** had a lower comprehensive effect. Specifically, α-M possessed a better performance on inhibiting Aβ fibrillogenesis, disaggregation, uptake and degradation than Zcbd-**2**, which was structurally modified at the C-8 position. This result indicated that the structure modification at the C-8 position might harm the activity of α-M. Compared to Zcbd-**2**, Zcbd-**3** exerted more substantial effects on Aβ disaggregation, microglial uptake and microglial degradation, and displayed similar features as α-M in all assessments.

On account of that, the main difference between Zcbd-**2** and Zcbd-**3** was the chemical groups at the C-2 position, and we hypothesized that it was a potential modification site. Moreover, the introduction of hydroxyl at the C-2 position counteracted the activity loss of the structural modification at the C-8 position, which suggested that the double hydroxylation at the C-2 position may enhance the multifunctional bioactivities of α-M. Besides, compared to Zcbd-**3**, the active effects of Zcbd-**4** and Zcbd-**5** were attenuated, which indicated that different modifications at the C-2 position would have different consequences for the bioactivity of α-M. Also, although Zcbd-**2**, Zcbd-**4** and Zcbd-**5**’s all-around performance was not as satisfactory as Zcbd-**3**, their performance in a specific aspect could be even better than α-M.

In conclusion, our study demonstrated the multifunctional features of α-M and its analogues, beneficial to Aβ fibrillogenesis, disaggregation, uptake and degradation, Aβ oligomer induced neurotoxicity, LPS induced neuroinflammation, and the integrity of tight junctions. It was found that the 3-methyl-2-butenyl group at C-8 is essential for the bioactivity of α-M, while modifying the double hydroxylation at the C-2 position may improve the multifunctional anti-AD effects. These structural insights may help us better understand the structure-activity relationship and accelerate α-M modification in drug development.

## Data Availability

The original contributions presented in the study are included in the article/Supplementary Material, further inquiries can be directed to the corresponding authors.

## Abbreviations

Aβ, amyloid-β; α-M, α-mangostin; AD, Alzheimer’s disease; BBB, blood-brain barrier; CCK-8, cell counting kit-8; IL-6, interleukin-6; NVU, the neurovascular unit; ThT, Thioflavin T; TJ, tight junction; TNF-α, tumour necrosis factor-α; ZO-1, Zona Occluden-1.
