# Supplementary figures and images for: Multifunctional Anti-Alzheimer’s Disease Effects of Natural Xanthone Derivatives: A Primary Structure-Activity Evaluation
Source: Front Chem. 2022 May 11;10:842208. doi: 10.3389/fchem.2022.842208 (PMC9130743; doi:10.3389/fchem.2022.842208)

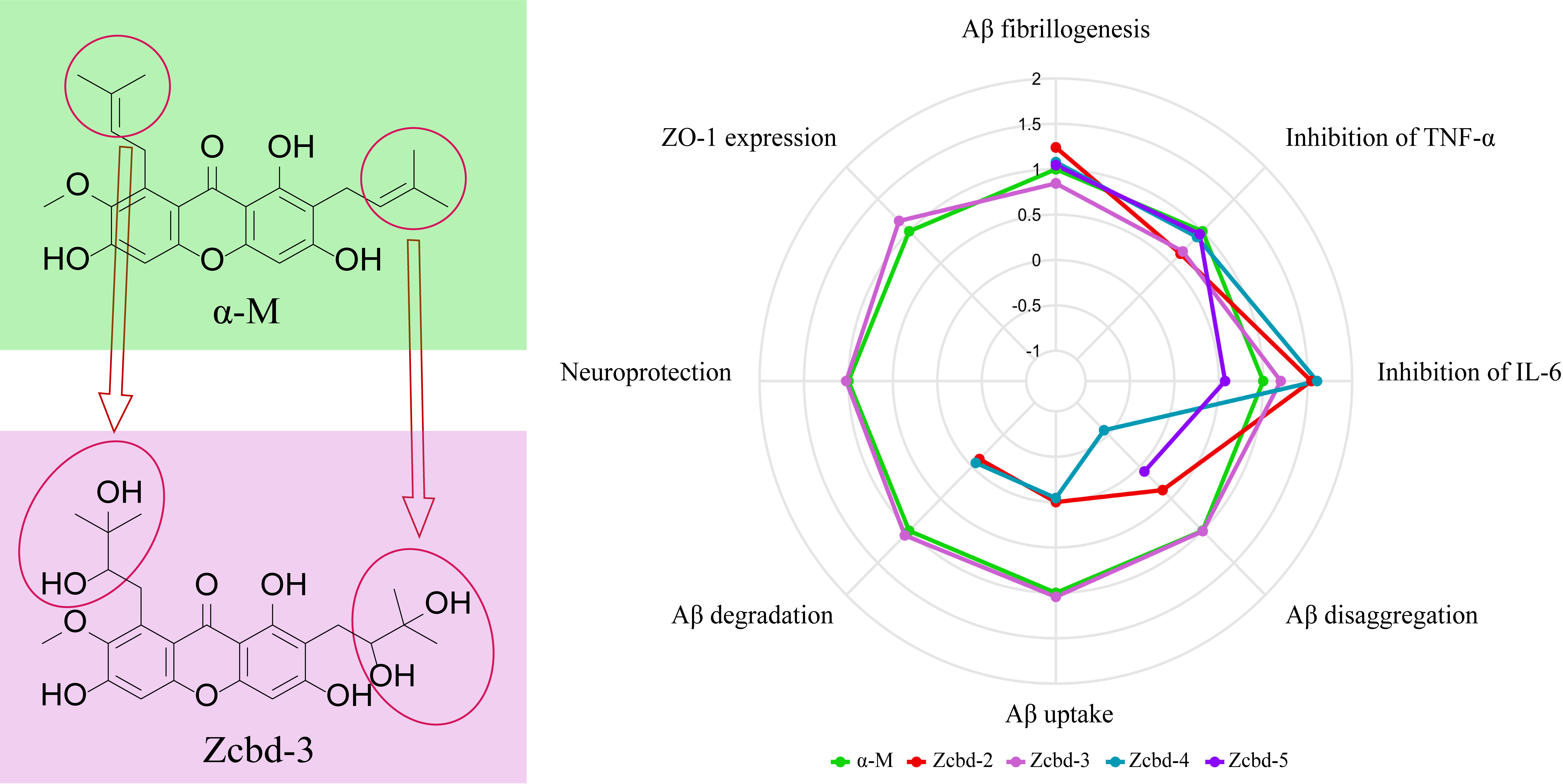

Supplement: Supplementary file 1 [file Image1.TIF]
